# Glycosylation profiling of triple-negative breast cancer: clinical and immune correlations and identification of LMAN1L as a biomarker and therapeutic target

**DOI:** 10.3389/fimmu.2024.1521930

**Published:** 2025-01-10

**Authors:** Qianru Yu, Hanyi Zhong, Xinhao Zhu, Chang Liu, Xin Zhang, Jiao Wang, Zongyao Li, Songchang Shi, Haoran Zhao, Cixiang Zhou, Qian Zhao

**Affiliations:** ^1^ Key Lab of Cell Differentiation and Apoptosis of Ministry of Education, Shanghai Jiao Tong University School of Medicine, Shanghai, China; ^2^ Ruijin Hospital, Shanghai Jiao Tong University School of Medicine, Shanghai, China; ^3^ Shanghai Jiao Tong University School of Medicine, Shanghai, China

**Keywords:** TNBC, glycosylation, machine learning, prognosis, tumor immune microenvironment

## Abstract

**Introduction:**

Breast cancer (BC) is the most prevalent malignant tumor in women, with triple-negative breast cancer (TNBC) showing the poorest prognosis among all subtypes. Glycosylation is increasingly recognized as a critical biomarker in the tumor microenvironment, particularly in BC. However, the glycosylation-related genes associated with TNBC have not yet been defined. Additionally, their characteristics and relationship with prognosis have not been deeply investigated.

**Methods:**

Transcriptomic analyses were used to identify a glycosylation-related signature (GRS) associated with TNBC prognosis. A machine learning-based prediction model was constructed and validated across multiple independent datasets. The model's predictive capability was extended to evaluate the prognosis of TNBC individuals, tumor immune microenvironment and immunotherapy response. *LMAN1L* (Lectin, Mannose Binding 1 Like) was identified as a novel prognostic marker in TNBC, and its biological effects were validated through experimental assays.

**Results:**

The GRS showed significant prognostic relevance for TNBC patients. The risk model effectively predicted molecular features, including immune cell infiltration and potential responses to immunotherapy. Experimental validation confirmed *LMAN1L* as a novel glycosylation-related prognostic gene, with low expression significantly inhibiting TNBC cell proliferation and migration.

**Discussion:**

Our GRS risk model demonstrates robust predictive capability for TNBC prognosis and immunotherapy response. This model offers a promising strategy for personalized treatment and improved clinical outcomes in TNBC.

## Introduction

Breast cancer (BC) is the most common malignant tumor in women (1), with triple-negative breast cancer (TNBC) accounting for 15-20% of BC cases. TNBC is characterized by poor prognosis, high recurrence, and limited treatment options, including surgery, chemotherapy, and radiotherapy (2, 3). Despite advances, metastatic TNBC has a median overall survival of less than two years, highlighting the need for improved therapies (4, 5).

Glycosylation, a crucial post-translational modification, plays a key role in cellular functions such as protein stability, immune evasion, and signal transduction (6–8). Tumor cells exhibit abnormal glycosylation patterns, which contribute to cancer progression, metastasis, and drug resistance (9–13). In BC, altered glycosylation affects cell signaling, adhesion, and immune recognition, promoting tumor aggressiveness (14–17). Suppressing protein glycosylation levels has also been shown to reduce drug resistance in malignant BC cells (18, 19). Presently, biomarkers for TNBC prognosis and therapy lack accuracy and suitability. However, glycosylation patterns in TNBC offer promising biomarkers for early diagnosis, prognosis, and treatment monitoring, as well as new therapeutic targets, though further in-depth research is essential to realize their full clinical potential.

Our study confirmed the value of glycosylation in TNBC, defined a set of glycosylation-related genes affecting TNBC prognosis, and used various machine learning algorithms to construct a clinical prediction model that can assist in predicting clinical prognosis and treatment, as well as evaluating the associated immune environment, responsiveness to immunotherapy, and the targeted drugs for high- and low-risk groups. Additionally, we conducted a series of experimental validations and preliminarily demonstrated its function and value, and innovatively discovered the potential of the glycosylation-related gene *LMAN1L* in TNBC.

## Materials and methods

### Data collection and preprocessing from public databases

RNA-seq data and corresponding clinicopathological information for the SCANB cohort obtained sourced from Mendeley Data (https://data.mendeley.com/datasets/yzxtxn4nmd/3) (20, 21). Additionally, genome-wide expression data and clinicopathological information for two other TNBC cohorts were retrieved from The Cancer Genome Atlas (TCGA-TNBC) (22) and Gene Expression Omnibus (GSE103091) (23, 24). RNA-seq data for BC cell lines were obtained from the Cancer Cell Line Encyclopedia (CCLE, https://sites.broadinstitute.org/ccle/) (25). Complete RNA-seq data and clinical characteristics for the IMvigor210 cohort, which focuses on anti-PD-L1 immunotherapy in bladder cancer, were retrieved from (http://research-pub.gene.com/IMvigor210CoreBiologies/). RNA-seq read counts from SCANB, TCGA, and GSE103091 were converted to transcripts per kilobase million (TPM) and subsequently log2-transformed. Similarly, RNA-seq read counts from the IMvigor201 cohort were converted to TPM and log2-transformed.

### Identification of glycosylation-related signature in TNBC

Differentially expressed genes (DEGs) were identified (p < 0.05, log2FC > 1) using the ‘DESeq2’ package in R (26), comparing TNBC tumor samples with normal breast tissue samples from the TCGA database. Prognosis-related genes (PRGs) were identified in all three cohorts (SCANB, TCGA, and GSE103091) via univariate Cox regression analysis (p < 0.05). Glycosylation-related genes (GRGs) were sourced from the KEGG BRITE Database (https://www.kegg.jp/kegg/brite.html) and previous literature (27), with the complete list provided in **Supplementary Table S1**. A total of 35 glycosylation-related genes that were both differentially expressed and prognostically significant were designated as glycosylation-related signature (GRS) for TNBC. The complete list of GRS is provided in **Supplementary Table S2**. The molecular pathways related to these genes were analyzed using the ‘clusterProfiler’ package (28) in R for Gene Ontology (GO) and Kyoto Encyclopedia of Genes and Genomes (KEGG) enrichment analysis.

### GRS risk model development using integrated machine learning techniques

To develop a high-accuracy, stable GRS risk model, we applied ten machine learning algorithms and 101 algorithmic combinations, including Lasso, Ridge regression, stepwise Cox regression, Elastic net (Enet), CoxBoost, random survival forest (RSF), supervised principal components (SuperPC), partial least squares regression for Cox (plsRcox), generalized boosted regression modeling (GBM), and survival support vector machine (survival-SVM).

The signature development process involved: (a) applying 101 algorithmic combinations to the GRS to construct prediction models based on leave-one-out cross-validation (LOOCV) within the SCANB cohort; (b) evaluating all models across the SCANB training dataset and two validation datasets (TCGA-TNBC and GSE103091); and (c) selecting the optimal model based on Harrell’s concordance index (C-index) across the validation datasets. The optimal model was defined using regression coefficients, with the GRS risk score formula as GRS risk score = $\sum_{i=1}^{n}Coefi*\left(expression\;of\;mRNAi\right)$ . Survival modeling and Kaplan-Meier (KM) analyses were performed across all datasets using the ‘survival’ and ‘survminer’ packages in R.

### Clinical and molecular significance of the GRS

Patients in the SCANB cohort were divided into high- and low-risk groups based on the median GRS risk score. Using PAM50 subtyping, the SCANB and TCGA cohorts were categorized into basal and non-basal groups. Differences in clinicopathological features between the two groups were compared across both the training and validation datasets and visualized using the ‘ggplot2’ package in R. Molecular pathways associated with DEGs between the two groups [p < 0.05, log2FC > 1, screened using ‘limma’ package in R (29)] further explored through GO and KEGG pathway analysis. Additionally, the GSEA software (https://www.gsea-msigdb.org/gsea/login.jsp/) was employed to analyze significantly enriched pathways of these DEGs.

### Evaluation of gene somatic mutations

Somatic mutation and copy number variation data for the SCANB cohort were retrieved (30). The ‘maftools’ R package (31) was used to analyze and visualize MAF files of somatic mutation data for the two groups and calculate the tumor mutation burden (TMB) score for SCANB patients.

### Tumor microenvironment immunological characteristics analysis

The immune cell infiltration for TNBC patients in the SCANB cohort was estimated using CIBERSORT (32), XCELL (33), and TIMER (34). The immune, stromal, and ESTIMATE scores, along with tumor purity scores, were calculated using ‘estimate’ package in R (35). Immunomodulators, including major histocompatibility complex (MHC) molecules, immunostimulators, immunostimulatory receptors, immunoinhibitors, and immunoinhibitory receptor markers, were collected from a previous study (36). The results were visualized using stacked graphs, heat maps, violin plots, and box plots generated with ‘ggplot2’ package in R. The Hematoxylin-eosin (HE) staining immunophenotype pathology image data (Formalin-fixed paraffin-embedding) in TCGA datasets were obtained from the Cancer Digital Slide Archive (CDSA, https://cancer.digitalslidearchive.org/).

### Prediction of therapeutic response

The SubMap algorithm (https://cloud.genepattern.org/gp) was employed to estimate the potential response of SCANB samples to immunotherapy. Predicted responses to anti-PD-1 and anti-CTLA-4 therapies were compared between high- and low-risk groups using annotation file subtype data (37). Additionally, the GRS risk model was applied to the IMvigor210 cohort to explore its predictive value for anti-PD-L1 therapy. Patients exhibiting stable disease (SD) or progressive disease (PD) were categorized as non-responders, whereas those showing complete response (CR) or partial response (PR) were categorized as responders. To validate the predictive robustness of the GRS risk model, we also analyzed a cohort of 144 melanoma patients treated with anti-PD-1 immune checkpoint blockade (ICB) (38).

### Prediction of potential drugs

Expression profile data of human cancer cell lines (CCLs) were downloaded from the Broad Institute Cancer Cell Line Encyclopedia (CCLE) project (39). Drug sensitivity analysis was performed using estimated AUC and IC50 values from the Genomics of Drug Sensitivity in Cancer (GDSC) database (40). Using the ‘lolR’ package in R, a nearest centroid classifier was developed to predict the risk group classification of BC cell lines based on the expression of essential genes from the SCANB cohort. Prior to drug sensitivity comparison, missing AUC values were imputed using K-nearest neighbor (k-NN) imputation, with compounds having over 20% missing data excluded. The intersections of potential drug candidates and comparisons between high- and low-risk groups were visualized using Venn diagrams, box plots, and bar plots created with ‘ggplot2’ package in R.

### Cell lines and cell culture

MDA-MB-231 cells were cultured in Leibovitz L-15 medium with 10% fetal bovine serum (FBS, Sigma, F2442). The LM2 cell line, derived from MDA-MB-231, was cultured in Dulbecco’s modified Eagle’s medium (DMEM) with 10% FBS. SUM159PT cells were maintained in Ham’s F12 medium supplemented with 5% FBS, 5μg/mL insulin, 1μg/mL hydrocortisone, and 10mM HEPES. MCF-10A cells were maintained in DMEM/F12 medium supplemented with 5% HS, 10μg/mL insulin, 0.5μg/mL hydrocortisone, 20ng/ml EGF, and 100ng/ml Cholera toxin. Cell lines BT-549, SUM149PT, and HCC1937 were cultured in RPMI 1640 with 10% FBS. All cell lines were cultured in a humidified 5% CO2 atmosphere at 37°C, except MDA-MB-231, which was maintained in 100% air. All cell lines were authenticated by short tandem repeat (STR) profiling.

### Cell transfection and Oligo RNA transfection

The sequences of small interfering RNA (siRNA) oligonucleotides (provided in **Supplementary Table S3**) were purchased from Shanghai GenePharma Co. Ltd. Oligo RNA transfection (100nM) was performed using Lipofectamine 2000 following the Reverse Transfection Protocol.

### RNA extraction, reverse transcription PCR and quantitative real-time PCR

Total RNA was extracted using TRIzol reagent (Invitrogen, Carlsbad, CA) following the manufacturer’s instructions. First-strand cDNA was synthesized using a Reverse Transcriptase kit (Tiangen, China). Quantitative Real-Time PCR (RT-qPCR) was performed using the SYBR Green method (Applied Biosystems, USA) on the 7900 Real-Time PCR System with the SDS 2.4 software sequence detection system (Applied Biosystems, USA). Primer sequences are listed in **Supplementary Table S4**. β-actin was used as an internal control to quantify mRNA levels. The relative expression levels of RNA were calculated using the 2−ΔΔCT method.

### Transwell migration assay

Cell migration ability was assessed using Corning transwell insert chambers (8μm pore size; Corning). A chemoattractant (600μl of medium containing 10% FBS) was added to the lower well of each chamber. Approximately 1.5 × 10^4 cells were seeded into each chamber and incubated for 20–22 hours at 37°C.

### Plate colony-forming assay

For plate colony-forming assay, cells were seeded into 3.5 cm culture dishes (800 cells per dish) and incubated for 7–12 days, with the medium changed every 3 days. The colonies were stained with crystal violet (1.5%, w/v; Sigma, St Louis, MO, USA).

### CCK-8 cell proliferation assay

For the CCK-8 assay, cells were seeded in 96-well plates (3 × 10^3 cells per well) and incubated lasting 3 days at 37°C. The changes of cell proliferation were monitored daily using CCK-8 reagent (Dojindo, Kumamoto, Japan), and the absorbance values were measured at 450 nm using a Hybrid Reader (BioTek, Winooski, VT, USA).

### Ethynyl-2’-deoxyuridine incorporation assay

Cells were seeded in 6-well plates at a density of 3×10^4 cells per well and cultured for 24 hours. Subsequently, 5μM EdU (Meilunbio, Dalian, China) was added and incubated for 2 hours. The remaining steps, including fixation, glycine incubation, and dilution, were carried out following standard protocols in a light-protected environment. Fluorescence images were captured using a fluorescent cell imager (Bio-Rad, Hercules, USA), and at least three random fields per well were photographed.

### Immunoblotting

Cells were lysed in 1× SDS lysis buffer for 15 minutes. Proteins were separated via SDS-PAGE and transferred to nitrocellulose membranes (Axygen, Union City, CA). After blocking with 5% non-fat milk, membranes were incubated overnight at 4°C with primary antibodies, followed by incubation with horseradish peroxidase-conjugated secondary antibodies. Signals were detected using an ECL detection kit (Millipore) and visualized using the ImageQuant LAS 4000 mini system (GE Healthcare, Piscataway, NJ, USA).

### Tissue samples and immunofluorescence

TNBC patient tissue samples were obtained from our previous study (41). Tissue sections (4-5 µm) were cut from paraffin-embedded blocks and mounted on slides. The slides were deparaffinized in xylene and rehydrated through a graded ethanol series. Antigen retrieval was performed by heating the slides in citrate buffer (pH 6.0) in a microwave for 20 minutes. Endogenous peroxidase activity was blocked with 3% hydrogen peroxide for 10 minutes. After blocking non-specific binding sites with normal serum, the sections were incubated overnight at 4°C with the primary antibody, followed by incubation with a fluorescent secondary antibody. The slides were then mounted and analyzed using microscopy.

### Statistical analysis

All statistical analyses were performed using R software (version 3.6.0) or GraphPad Prism version 10 software. Significance was calculated using unpaired two-tailed Student’s t test. Data are represented as mean ± SEM or ± SD with at least three independent experiments. For all figures, statistical significance was represented as *P < 0.05, **P < 0.01, and ***P < 0.001.

## Result

### Construction of a glycosylation-related signature

**Figure 1:**
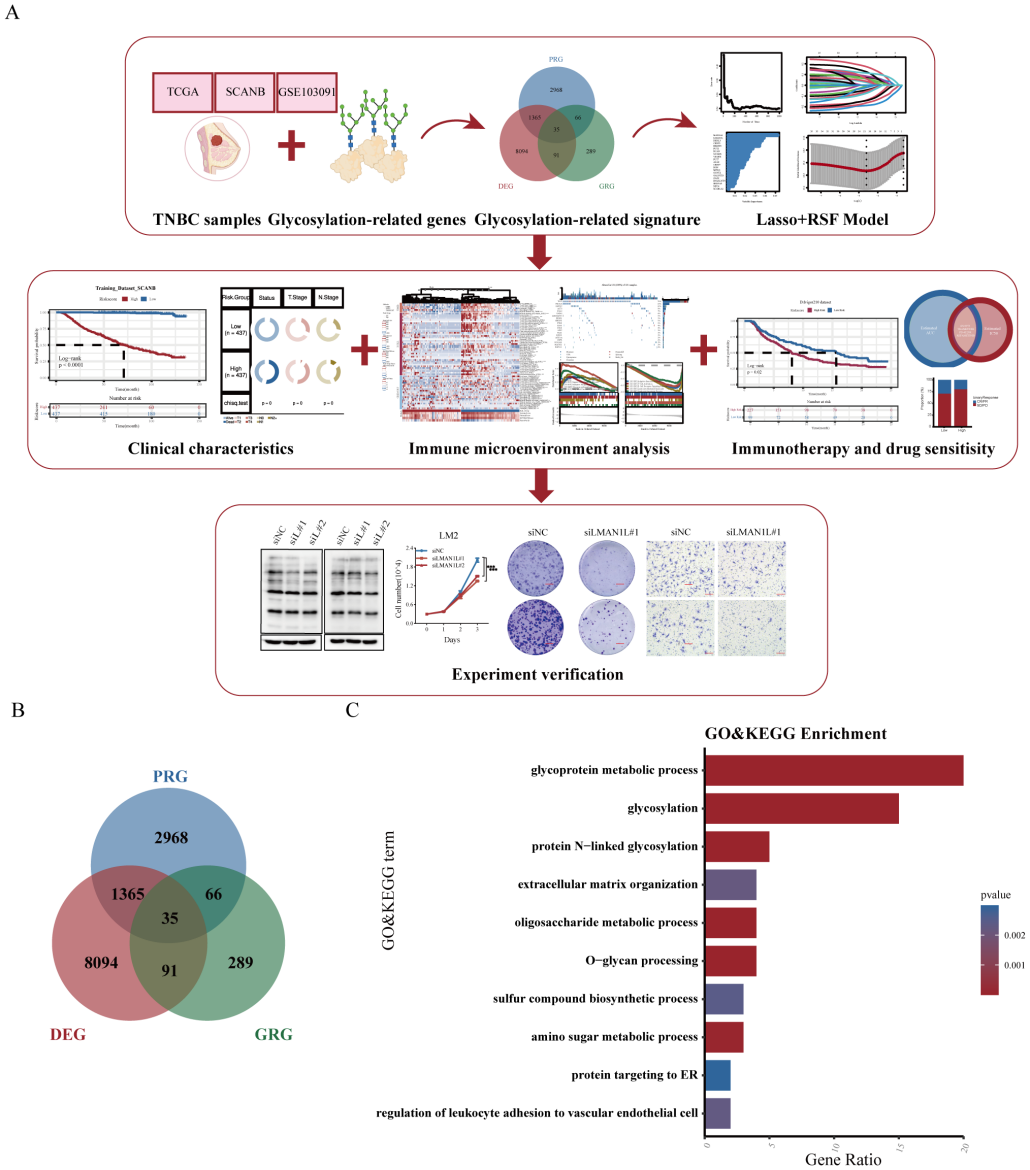
Construction of a glycosylation-related signature. **(A)** Study flow chart. **(B)** Venn diagram showing the intersection of prognosis-related genes (PRG), differentially expressed genes (DEG), and glycosylation-related genes (GRG). **(C)** Gene Ontology (GO) and Kyoto Encyclopedia of Genes and Genomes (KEGG) pathway analysis of the glycosylation-related signature (GRS).

The flowchart of our study was shown in **Figure 1A**. Initially, based on the expression profiles of three cohorts (SCANB, TCGA-TNBC, and GSE103091), we identified 4,434 prognostic-related genes (PRGs) through univariate Cox regression analysis. In addition, 9,589 differentially expressed genes (DEGs) were identified between TNBC tumor samples and adjacent normal tissues with statistical significance (p < 0.05 and log2FC > 1). We then integrated glycosylation-related genes from the KEGG BRITE Database and a previous study (27), resulting in a gene set of 481 glycosylation-related genes. By intersecting the three gene sets, we identified a glycosylation-related signature (GRS) with prognostic significance in TNBC (**Figure 1B**). Further Gene Ontology (GO) and Kyoto Encyclopedia of Genes and Genomes (KEGG) enrichment analyses revealed that the GRS is involved in multiple glycosylation-associated pathways, including glycoprotein metabolic processes, protein N-linked glycosylation, O-glycan processing, oligosaccharide metabolic process and amino sugar metabolic process (**Figure 1C**).

### Construction of a GRS risk model via machine learning algorithms

**Figure 2:**
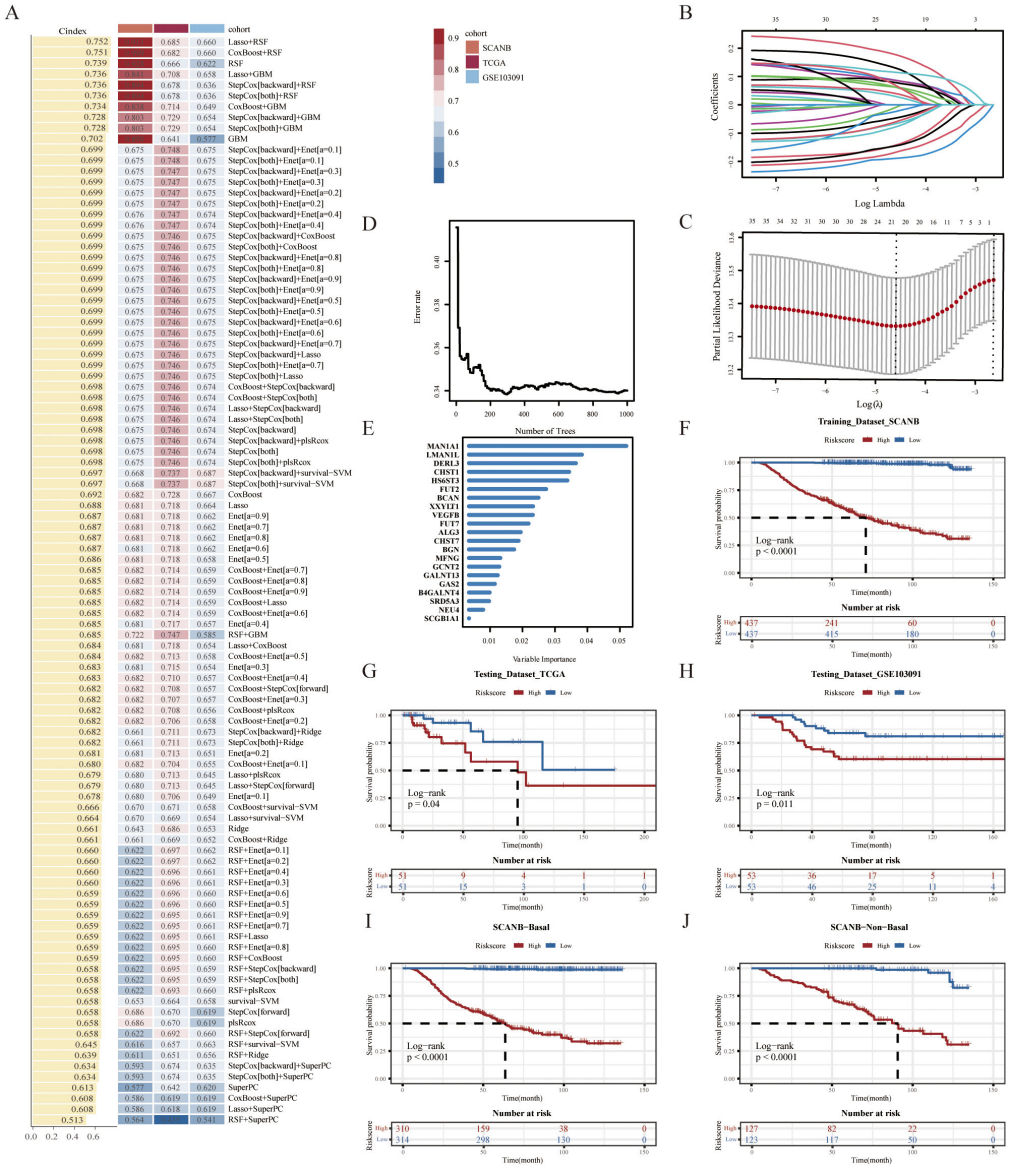
Construction of a GRS risk model via machine learning algorithms. **(A)** 101 prediction models were evaluated using the leave-one-out cross-validation (LOOCV) framework, with the concordance index (C-index) calculated for each model across all validation datasets. **(B)** The optimal λ value in the SCANB cohort was identified at the minimum partial likelihood deviance. **(C)** Lasso coefficients were derived for the most informative prognostic genes. **(D)** Error rate and **(E)** variable importance were assessed using random survival forest (RSF) analysis. Kaplan-Meier survival curves for overall survival (OS) were stratified by GRS risk groups in **(F)** SCANB, **(G)** TCGA-TNBC, **(H)** GSE103091, **(I)** SCANB-Basal and **(J)** SCANB-Non-Basal cohorts.

To ensure high accuracy and stability, we utilized multiple machine learning algorithms to develop a risk model using GRS. In the SCANB dataset, 101 prediction models were fitted using the leave-one-out cross-validation (LOOCV) framework, and the C-index of each model was calculated across all validation datasets (**Figure 2A**). Notably, the optimal model combined Lasso regression with the Random Survival Forest (RSF), achieving the highest average C-index (0.752). This combination consistently outperformed other models in all validation datasets. In the Lasso regression, the optimal λ was determined when the partial likelihood deviance minimized, based on the LOOCV framework (**Figures 2B, C**). Using these two algorithms, we performed gene selection and model construction, identifying the 21 genes with the highest variable importance (**Figures 2D, E**). A risk score for each patient was then calculated using the expression levels of the 21 genes, weighted by their regression coefficients. Patients were stratified into high- and low-risk groups based on the optimal cut-off value, determined by the ‘survminer’ package. Patients in the high-risk group had significantly worse overall survival (OS) compared to those in the low-risk group across the SCANB training dataset and validation datasets (all P < 0.05) (**Figures 2F–H**). Using the basal-like phenotype, we categorized TNBC patients into basal and non-basal groups and observed that our model maintained consistent predictive accuracy across the subtypes (**Figures 2I, J**, **Supplementary Figures S1A, B**).

### Association of GRS risk model with clinical characteristics in TNBC patients

**Figure 3:**
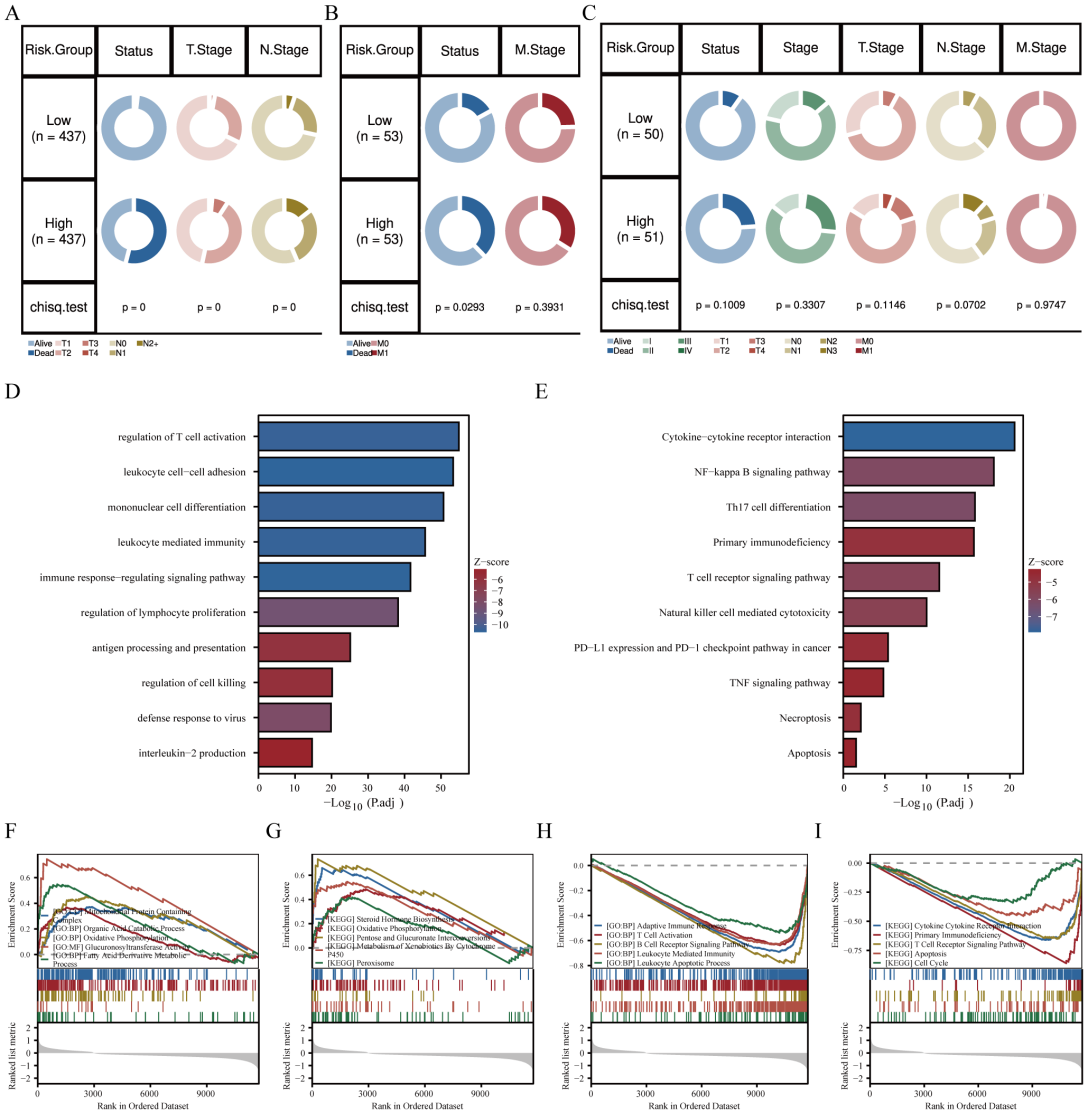
Association of GRS risk model with clinical characteristics in TNBC patients. Circos plots showing survival and pathological differences between two risk groups in the **(A)** SCANB, **(B)** GSE103091, and **(C)** TCGA-TNBC cohorts (Chi-square test). **(D)** GO and **(E)** KEGG analysis of differentially expressed genes between high- and low-risk groups. **(F-I)** GO and KEGG terms enriched by differentially expressed genes using GSEA analysis between the two risk groups.

In addition, we investigated the relationship between the risk score and clinical features. In the SCANB cohort, the results suggested a significant difference in overall stage, N stage, and T stage (**Figure 3A**). In the GSE103091 database, we also observed a significant correlation between risk scores and clinical staging (**Figure 3B**). Unfortunately, due to limitations in sample size in the TCGA database, we did not observe a significant difference in clinical characteristics between high- and low-risk groups. However, it can be seen that the patients in the high-risk group had more advanced tumor progression, which is consistent with the results of the other two databases (**Figure 3C**). Furthermore, we explored the biological functions and enriched pathways between low- and high-risk groups. The high-risk group was significantly enriched for regulation of lymphocyte activation, regulation of immune response and cytokine production by GO analysis (**Figure 3D**). KEGG analysis indicated that the top three pathways were cytokine-cytokine receptor interaction, NF-kappa B signaling pathway and Th17 cell differentiation (**Figure 3E**). To further confirm the correlation between risk scores and biological function, we performed Gene Set Enrichment Analysis (GSEA). The results showed that the risk score was positively correlated with oxidative phosphorylation and organic acid catabolic process (**Figures 3F, G**), and was negatively correlated with adaptive immune response, lymphocyte receptor signaling pathway, and cytokine-cytokine receptor interaction (**Figures 3H, I**).

### Correlation of immune microenvironment with GRS risk model

**Figure 4:**
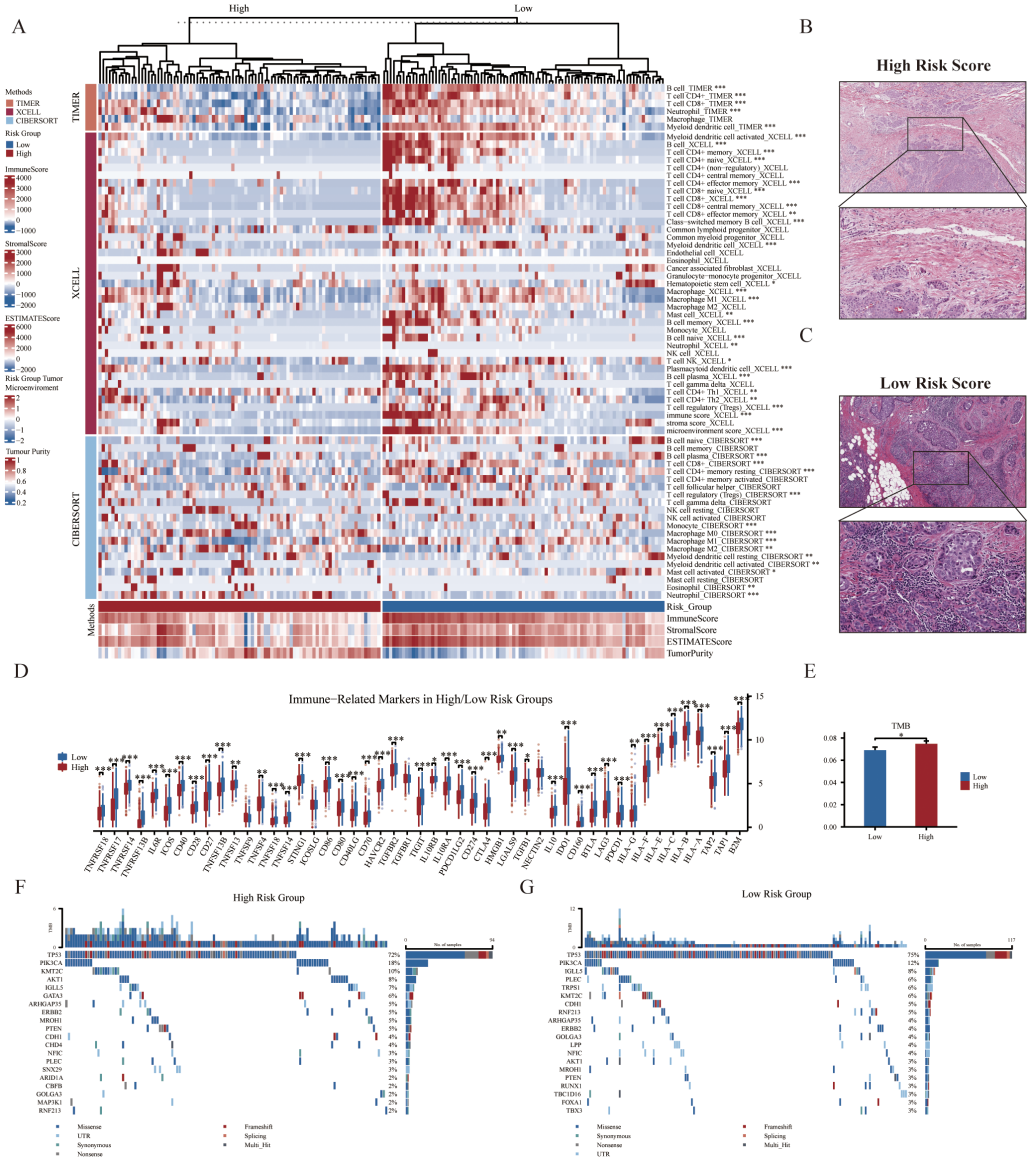
Correlation of immune microenvironment with GRS risk model. **(A)** Thermogram displaying relationships between GRS risk groups (top 20% samples) and tumor immune microenvironment components based on CIBERSORT, XCELL, TIMER, and ESTIMATE analyses (Wilcoxon test). The representative images show the variations in pathological HE staining between the **(B)** high- and **(C)** low-risk groups. **(D)** Boxplot illustrating the association between GRS risk groups and the mRNA expression levels of various immune-related markers (Wilcoxon test). **(E)** Histogram depicting differences in tumor mutation burden (TMB) between risk groups (Wilcoxon test). Waterfall plots of genetic alterations in common mutant genes for the **(F)** high- and **(G)** low-risk groups in the SCANB cohort. *P < 0.05, **P < 0.01, ***P < 0.001.

The above GSEA revealed that several immune-related pathways were highly enriched in the low-risk group, and we consequently investigated the immune landscape and expression of immune-related markers between the two groups. We employed a variety of algorithms, including TIMER, XCELL, and CIBERSORT to comprehensively investigate the infiltration of immune cells across two groups within the SCANB cohort (**Figure 4A**). We found that the low-risk group exhibited a relatively higher infiltration abundance of immune cell types, including B cells, CD4+ naïve T cells, CD8+ T cells and neutrophils (all p<0.05). However, the high-risk group exhibited lower immune cell infiltration, with a higher presence of tumor parenchymal cells. The HE staining immunophenotype also revealed that high-risk scores were associated with reduced immune cell infiltration, whereas low-risk scores were correlated with increased immune cell infiltration (**Figures 4B, C**). In addition, among the immune-related markers, the low-risk group had significantly higher relative expression levels, such as *NECTIN2*, *CD70*, *TNFSF4*, *TNFSF14*, *TNFSF18*, and *CD27* (**Figure 4D**). In terms of genomic heterogeneity, we observed that the high-risk group had a higher tumor mutation burden (TMB) (**Figure 4E**). Further, after comparing the two groups of genes with high mutation frequencies we found that oncogene *PIK3CA* was relatively high mutated in the high-risk group and the classical tumor suppressor gene *TP53* was mutated at a higher frequency in the low-risk group (**Figures 4F, G**).

### Predictive value of GRS risk model for immunotherapy

**Figure 5:**
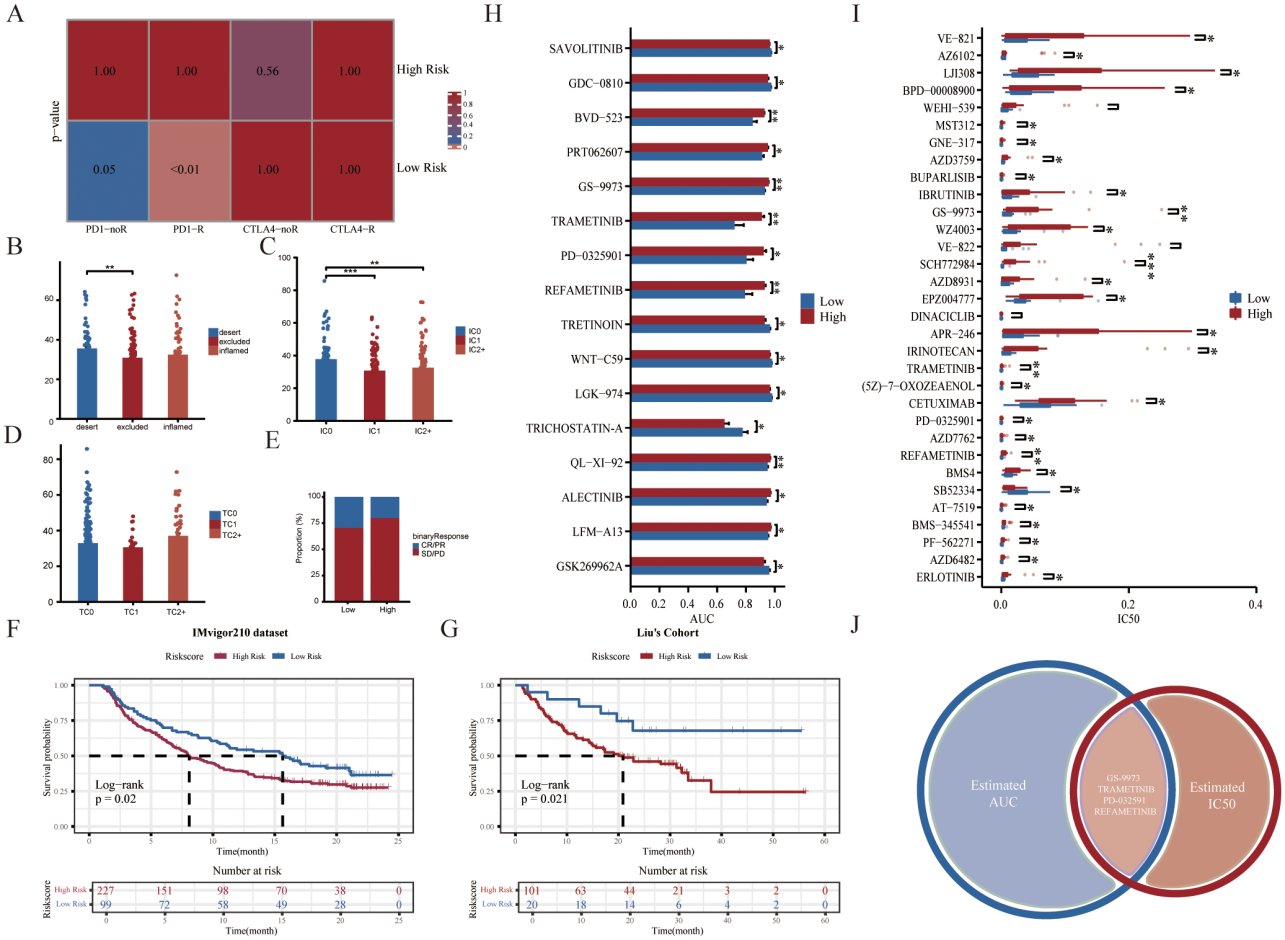
Predictive value of GRS risk model for immunotherapy and identification of potential therapeutic agents. **(A)** Prediction of immunotherapy responses between two GRS risk groups in the SCANB cohort. **(B)** Histogram showing differences in GRS scores across immune phenotypes in the IMvigor210 cohort (Kruskal-Wallis test). **(C, D)** Histograms displaying variations in PD-L1 expression across immune cell (IC) and tumor cell (TC) subsets in the IMvigor210 cohort (Wilcoxon test). **(E)** Stacked histogram of anti-PD-L1 responsiveness between GRS risk groups in the IMvigor210 cohort. Kaplan-Meier survival curves for GRS risk groups in the IMvigor210 cohort **(F)** and Liu’s cohort **(G)**. **(H)** Histogram of area under the curve (AUC) values between high- and low-risk groups from the GDSC dataset (Wilcoxon test). **(I)** Boxplot showing variations in IC50 values between risk groups from the GDSC dataset (Wilcoxon test). **(J)** Venn diagram showing compounds with significant differences in both AUC and IC50 values in the GDSC dataset. *P < 0.05, **P < 0.01, ***P < 0.001.

Given that patients in the low-risk group had higher levels of immune cell infiltration, we hypothesized that they would be more sensitive to immunotherapy. The results of the Subclass Mapping (Submap) suggested that the expression patterns of patients in the low-risk group were similar to those of TNBC patients who responded to anti-PD-1 immunotherapy (**Figure 5A**). In addition, to further explore the predictive efficacy of GRS for anti-PD-L1 therapies, the GRS risk model was applied to the IMvigor210 cohort. Similar to previous results, we observed a high-risk score in immune desert type and IC0 (lowest immune cell score) type (**Figures 5B, C**). We also examined differences in risk scores among the TC groups (**Figure 5D**). The low-risk group also showed better immunotherapy response rates and better prognosis (**Figures 5E–G**). Overall, these results demonstrated that the low-risk group was more likely to benefit from immunotherapy.

### Identification of potential therapeutic agents for the high-risk group

To explore the potential relationship between our GRS risk model and drug sensitivity, we analyzed half-maximal inhibitory concentration (IC50) values and AUC values for several drugs from the Genomics of Drug Sensitivity in Cancer (GDSC) database (**Figures 5H, I**). The results demonstrated that four compounds (including GS-9973, TRAMETINIB, PD-0325901, and REFAMETINIB) could be the potential therapeutic agents for the high-risk group (**Figure 5J**). Among them, TRAMETINIB, PD-0325901, and REFAMETINIB are all MEK inhibitors. Trametinib is the first FDA-approved MEK inhibitor. When taken alone or in combination with other treatments, MEK inhibitors have been shown to have good anti-tumor activity in melanoma, lung cancer, and colorectal cancer (42, 43). Thus, our study may provide guidance for the treatment of specific TNBC patient subgroups in the clinic.

### Validation of mRNA and protein expression in GRS risk model

**Figure 6:**
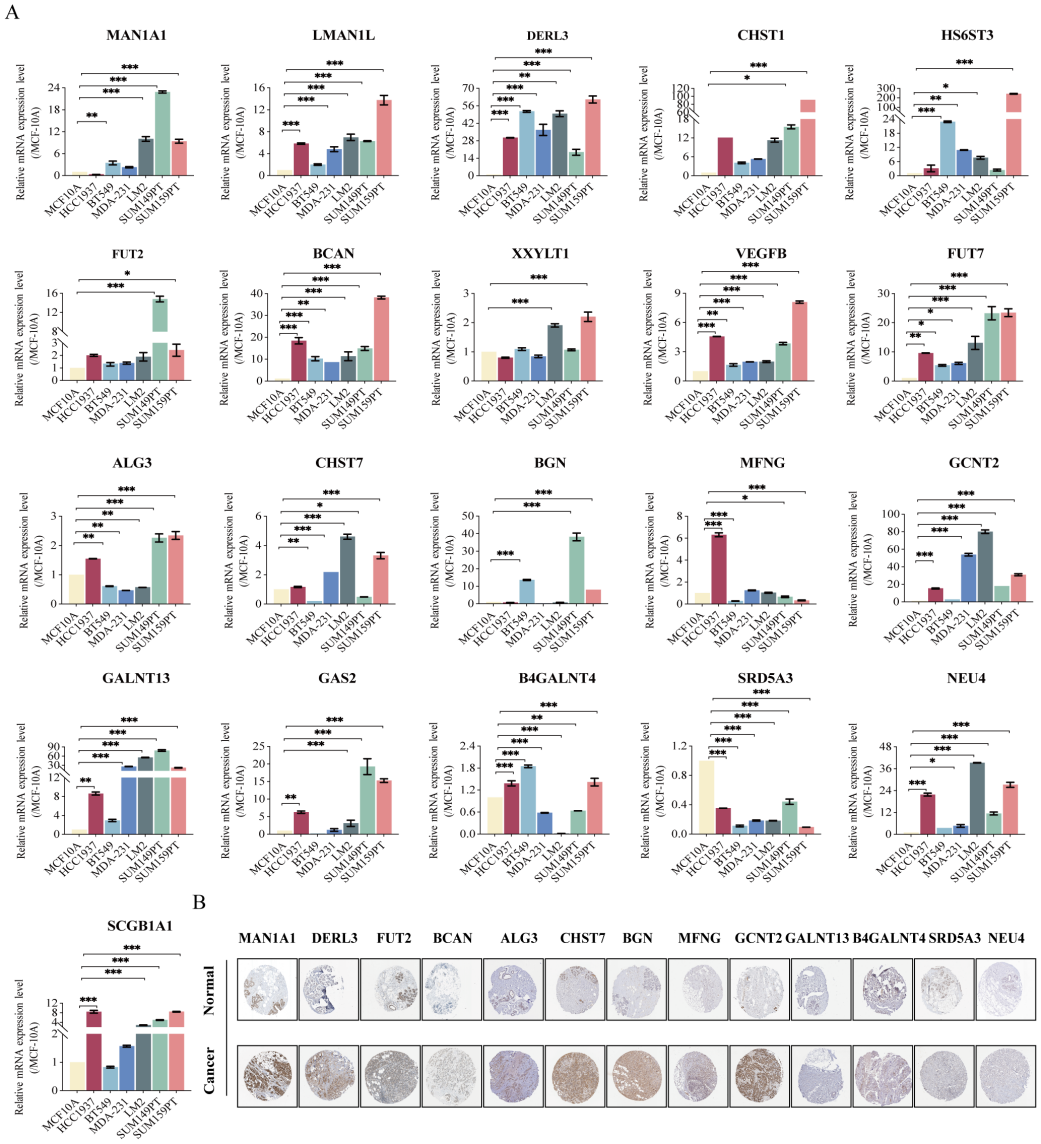
Validation of mRNA and protein expression in GRS risk model. **(A)** The expression of 21 gene mRNA in TNBC cell lines experimented by RT-qPCR analysis: *MAN1A1*, *LMAN1L*, *DERL3*, *CHST1*, *HS6ST3*, *FUT2*, *BCAN*, *XXYLT1*, *VEGFB*, *FUT7*, *ALG3*, *CHST7*, *BGN*, *MFNG*, *GCNT2*, *GALNT13*, *GAS2*, *B4GALNT4*, *SRD5A3*, *NEU4*, and *SCGB1A1*. **(B)** Protein expression in breast cancer and normal tissues validated by the immunohistochemistry analysis of the HPA database. ^*^P < 0.05, ^**^P < 0.01, ^***^P < 0.001.

We utilized RT-qPCR analysis to validate the differential mRNA expression of 21 model genes in TNBC cell lines (**Figure 6A**). Several genes were up-regulated in all six TNBC cell lines compared to MCF-10A, including *LMAN1L*, *DERL3*, *CHST1*, *HS6ST3*, *FUT2*, *BCAN*, *VEGFB*, *FUT7*, *GCNT2*, *GALNT13*, and *NEU4*. We also observed that the expression of two genes (*LMAN1L* and *NEU4*) was highest in the more malignant TNBC cell lines (LM2 and SUM159PT), suggesting that they may play a role in TNBC progression. *MAN1A1*, *CHST7*, *BGN*, *GAS2*, and *SCGB1A1* were also up-regulated in most TNBC cell lines. But, the gene *SRD5A3* showed a significant down-regulation in all TNBC cell lines. Additionally, we verified the expression of the model genes at the protein level between BC and adjacent normal tissues using the Human Protein Atlas (HPA) database (https://www.proteinatlas.org). The majority of genes showed higher staining in BC samples (**Figure 6B**).

### LMAN1L promotes the malignant characteristics of TNBC

**Figure 7:**
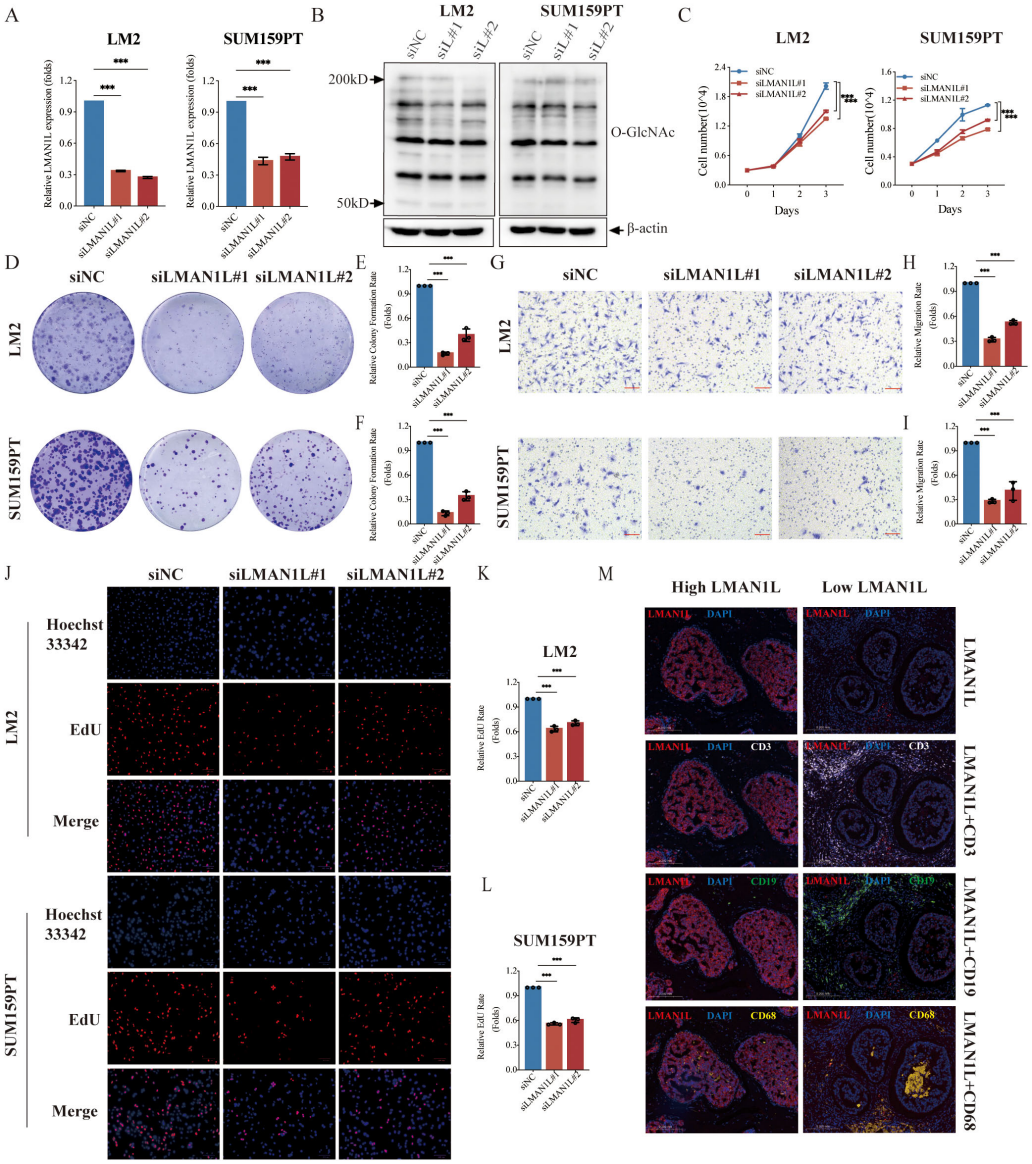
LMAN1L promotes the malignant characteristics of TNBC. **(A)** RT-qPCR analyzing the knockdown efficiency of *LMAN1L* in LM2 cells and SUM159PT cells. **(B)** The level of overall intracellular O-GlcNAcylation in LM2 cells and SUM159PT cells following treatment with siRNA sequences (siNC, si*LMAN1L*#1, si*LMAN1L*#2). **(C)** Cell proliferation assays using CCK-8 in LM2 cells and SUM159PT cells with *LMAN1L* knockdown. **(D)** Representative images (scale bar 0.5cm) and quantification **(E, F)** of plate colony formation assay. **(G)** Representative images (scale bar 100μm) and quantification **(H, I)** of transwell migration assay in LM2 cells and SUM159PT cells with *LMAN1L* knockdown. **(J)** Representative images and quantification **(K, L)** of EdU assay in LM2 cells and SUM159PT cells with *LMAN1L* knockdown. EdU (red) and Hoechst 33342 (blue). **(M)** Representative images of immunofluorescence assay in TNBC patient samples grouped based on LMAN1L protein expression. LMAN1L (red), DAPI (blue), CD3 (white), CD19 (green), and CD68 (yellow). ***P < 0.001.

Among the top five genes ranked in the GRS risk model, *MAN1A1* (44, 45), *DERL3* (46), *CHST1* (47), and *HS6ST3* (48) have all been reported for their roles in BC, whereas the role of *LMAN1L* in various cancers, especially TNBC, has not been investigated. *LMAN1L* ranks second in importance, making a major contribution to the model. The qPCR results also demonstrate a substantial difference in its expression between TNBC cell lines and normal breast epithelial cells. Therefore, we performed a series of *in vitro* experiments to examine the role of *LMAN1L* in TNBC. First, we silenced *LMAN1L* expression in LM2 and SUM159PT cells, which have high levels of *LMAN1L*, using two distinct *LMAN1L*-targeting small interfering RNAs (siRNAs), si*LMAN1L*#1 and si*LMAN1L*#2 (**Figure 7A**). Subsequently, we assessed the overall levels of intracellular O-GlcNAcylation after *LMAN1L* knockdown in these cells and observed that LMAN1L likely inhibits O-GlcNAcylation (**Figure 7B**). Compared to the NC group we also observed that inhibition of *LMAN1L* expression significantly suppressed the proliferation of LM2 and SUM159PT (**Figure 7C**). The plate colony-forming assay and transwell migration assay brought similar results, finding that knockdown of *LMAN1L* resulted in a significant attenuation of clone formation and migration of TNBC cells (**Figures 7D–I**). Additionally, we carried out further validation by EdU experiments (**Figures 7J–L**). We also analyzed immune cell infiltration in tumor samples from TNBC patients with different LMAN1L expression levels and found that high LMAN1L expression correlated with reduced immune cell infiltration (**Figure 7M**). In summary, we found that *LMAN1L* being a glycosylation-related gene may play an important role in TNBC. All data were presented as means with standard deviations from three independent experiments.

## Discussion

Glycosylation plays a critical role in tumor biology by influencing key processes such as cell adhesion, extracellular matrix interactions, and cell signaling. These processes are essential for tumor development, including invasion, angiogenesis, metastasis, and immune response regulation (49–51). In the tumor microenvironment (TME), abnormal glycosylation often contributes to immune evasion, leading to immune tolerance.

We observed significant differences in immune cell infiltration between high- and low-risk groups using the GRS risk model. Immune cell infiltration, including B cells, CD4+ naïve T cells, and CD8+ T cells, was higher in the low-risk group. Tumor-infiltrating lymphocytes (TILs) are increasingly recognized for their role in TNBC prognosis, and TME remodeling (recruitment of CD4+ T cells, CD8+ T cells, and NK cells) can enhance the efficacy of immunotherapy (52–55). These findings suggest that low-risk group patients may benefit more from immunotherapy. In the immunotherapy cohort, responders had lower risk scores, likely due to more effective immune responses, reinforcing that the GRS risk model can assess the tumor immune microenvironment and identify glycosylation as a potential biomarker for TNBC.

Our study also highlights the role of glycosylation-related genes in TNBC. *MAN1A1*, upregulated in most TNBC cell lines, participates in the maturation of Asn-linked oligosaccharides. Low expression of *MAN1A1* often leads to abnormal N-glycosylation and is associated with cell adhesion and brain metastasis in BC (44). *DERL3*, another gene highly expressed in TNBC cell lines, targets misfolded glycoproteins in the ER and is associated with lymph node metastasis (56) and poor prognosis (46). The expression of sulfotransferase *CHST1* is related to the sensitivity of Siglec ligands to carbohydrate sulfation inhibition, and its high expression is generally associated with poor prognosis in specific cancers (47). Notably, we identified *LMAN1L*, a glycosylation-related gene previously unreported in BC, which plays a role in TNBC malignancy. LMAN1L is a type I transmembrane protein involved in transporting glycoproteins from the ER to the Golgi apparatus. Our results show that inhibiting LMAN1L suppresses TNBC cell proliferation, clonogenicity, and migration, and modulates the immune microenvironment. High expression of *LMAN1L* is also associated with poor prognosis in pancreatic cancer (57). These findings suggest that LMAN1L may contribute significantly to TNBC progression, though further studies are needed to elucidate its precise mechanisms.

While our study provides valuable insights, several limitations must be addressed. TNBC is highly heterogeneous, with diverse histological phenotypes, necessitating more comprehensive mechanistic and clinical research on the roles of glycosylation-related genes in various TNBC subtypes. Although RT-qPCR confirmed differential mRNA expression of model genes in TNBC cell lines, protein expression remains unvalidated. Further investigation into the molecular mechanisms of glycosylation-related genes in TNBC is essential to fully understand their impact on tumor biology and treatment responses.

## Conclusion

We developed and validated a glycosylation-related risk model for prognostic stratification in TNBC patients. This model effectively predicts clinical outcomes and immunotherapy responses. Future research should explore the mechanisms of glycosylation-related genes in TNBC, potentially guiding novel therapeutic strategies.

## Data Availability

The original contributions presented in the study are included in the article/**Supplementary Material**. Further inquiries can be directed to the corresponding authors.
