# Supplementary material for: Glycosylation profiling of triple-negative breast cancer: clinical and immune correlations and identification of LMAN1L as a biomarker and therapeutic target
Source: Front Immunol. 2025 Jan 10;15:1521930. doi: 10.3389/fimmu.2024.1521930 (PMC11759290; doi:10.3389/fimmu.2024.1521930)
Supplement: Supplementary file 1 [file DataSheet1.pdf]

Supplementary Figures

A

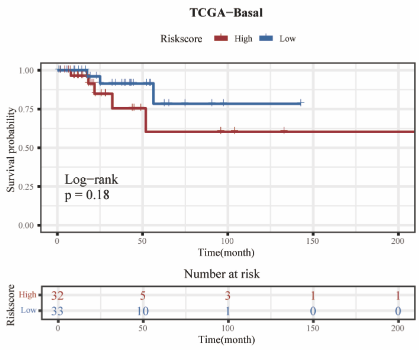

B

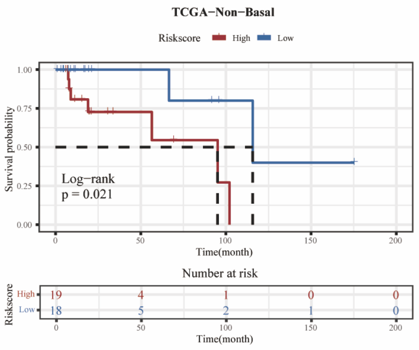

Figure S1. Kaplan-Meier survival curves for overall survival (OS) were stratified by GRS risk groups in (**A**) TCGA-Basal and (**B**) TCGA-Non-Basal cohorts.
